# Acute on Chronic Pancreatitis Causing a Highway to the Colon with Subsequent Road Closure: Pancreatic Colonic Fistula Presenting as a Large Bowel Obstruction Treated with Pancreatic Duct Stenting

**DOI:** 10.1155/2015/794282

**Published:** 2015-03-17

**Authors:** Justin Cochrane, Greg Schlepp

**Affiliations:** ^1^Junior Faculty Spokane Teaching Health Center (Internal Medicine Residency), Providence Sacred Heart Medical Center, Spokane, WA 99204, USA; ^2^Spokane Digestive Disease Center, Spokane, WA 99204, USA

## Abstract

*Context*. Colonic complications associated with acute pancreatitis have a low incidence but carry an increased risk of mortality with delayed diagnosis and treatment. Pancreatic colonic fistula is most commonly associated with walled off pancreatic necrosis or abscess formation and rarely forms spontaneously. Classic clinical manifestations for pancreatic colonic fistula include diarrhea, hematochezia, and fever. Uncommonly pancreatic colonic fistula presents as large bowel obstruction. *Case*. We report a case of a woman with a history of recurrent episodes of acute pancreatitis who presented with large bowel obstruction secondary to pancreatic colonic fistula. Resolution of large bowel obstruction and pancreatic colonic fistula was achieved with pancreatic duct stenting. *Conclusion*. Pancreatic colonic fistula can present as large bowel obstruction. Patients with resolved acute pancreatitis who have radiographic evidence of splenic flexure obstruction, but without evidence of mechanical obstruction on colonoscopy, should be considered for ERCP to evaluate for PCF. PCF not associated with walled off pancreatic necrosis or peritoneal abscess can be treated conservatively with pancreatic duct stenting.

## 1. Introduction

Pancreatic colonic fistula (PCF) occurs in 0.4 to 5% of patients with acute pancreatitis [1, 2]. Most cases of PCF formation occur secondary to percutaneous drainage or surgical debridement of pancreatic necrosis. Typical clinical manifestations are diarrhea, hematochezia, and fever [3]. Rarely does PCF present as large bowel obstruction. A 73-year-old female with acute on chronic pancreatitis developed large bowel obstruction secondary to spontaneous PCF formation.

## 2. Case

**Figure 1:**
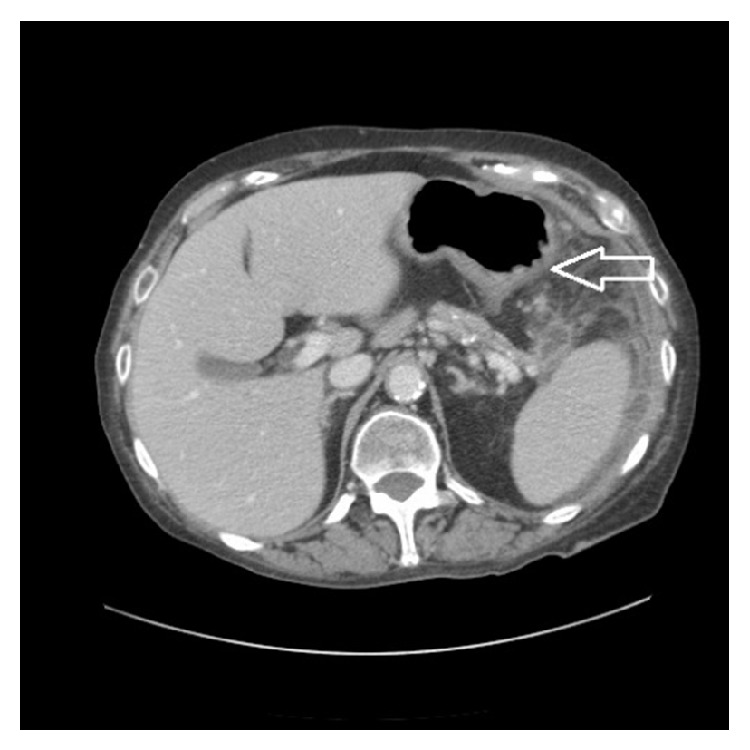
CT abdomen/pelvis done at time of admission to outside hospital showing colonic thickening at the splenic flexure.

**Figure 2 fig2:**
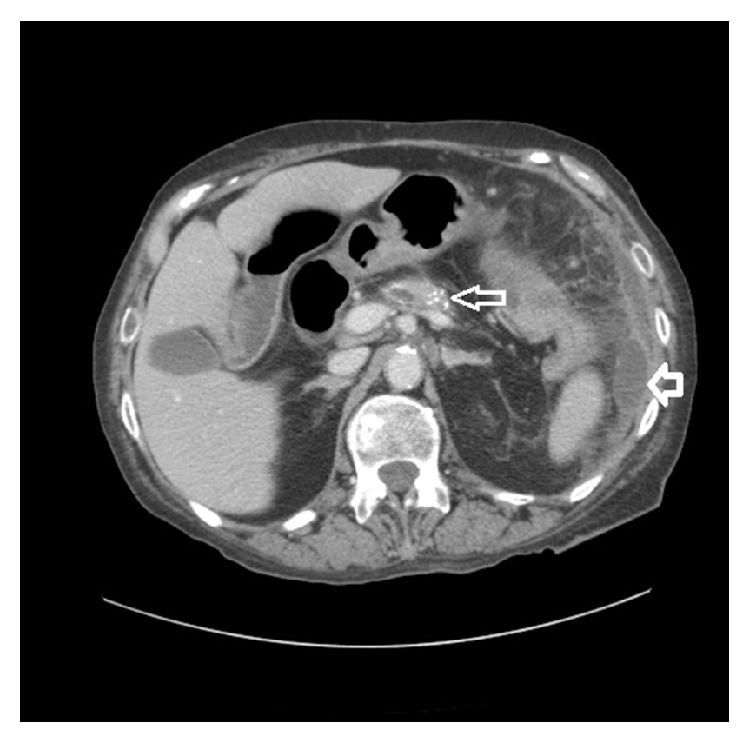
CT abdomen/pelvis done at outside hospital at time of admission: Fat arrow indicates fluid collection near the pancreatic tail and splenic flexure 3.5 cm. Skinny arrow calcification of the pancreas suggesting chronic pancreatitis.

**Figure 3:**
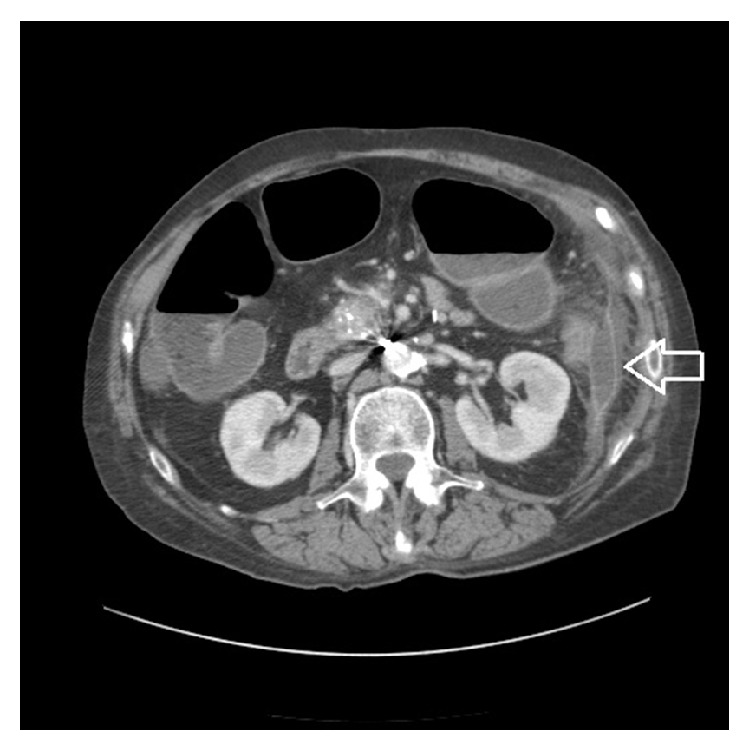
CT abdomen/pelvis done at outside hospital at time of admission showing fluid collection around the pancreatic tail and splenic flexure 4.7 cm.

**Figure 4:**
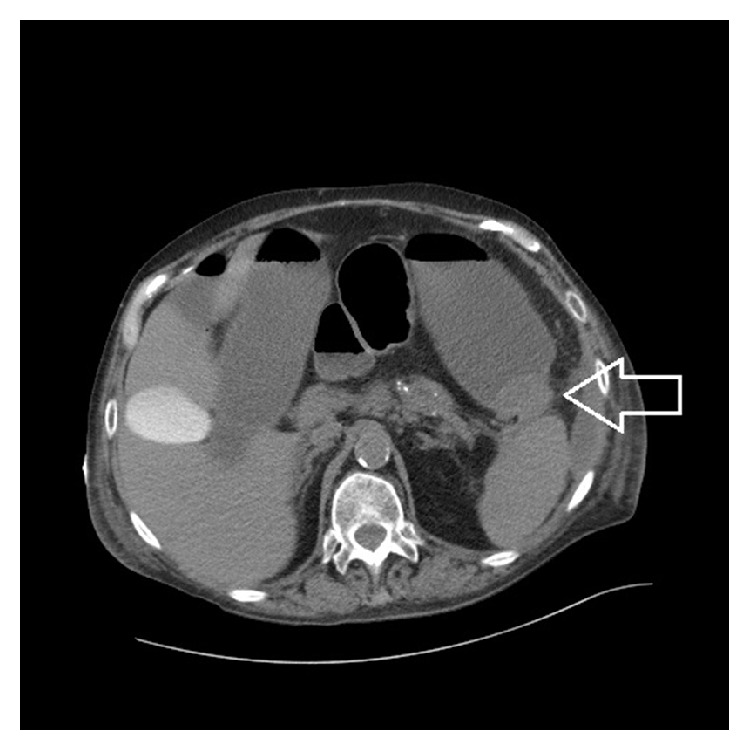
CT abdomen/pelvis done at admission to tertiary medical center demonstrating cut-off sign at the splenic flexure with distended transverse colon.

Mrs. L is a 73-year-old female with a history of chronic pancreatitis secondary to chronic alcohol abuse who presented to an outside hospital with worsening abdominal pain and distention. At the outside hospital she was diagnosed with pancreatitis. She received pain medication, intravenous fluids, and bowel rest. She demonstrated no signs of clinical improvement with conservative medical management over the next 48 hours. CT imaging of the abdomen/pelvis demonstrated inflammatory stranding around the splenic flexure of the colon and two small fluid collections (3.6 cm and 4.7 cm) adjacent to pancreatic tail and splenic flexure (Figures 1 [Fig fig2]–3). No mechanical obstruction was noted on colonoscopy. Abdominal pain and distention improved after colonoscopy but returned over the next 24 hours. Repeat CT imaging 4 days later demonstrated high grade obstruction of the large bowel with stricture at the splenic flexure with fluid collections remaining stable and resolution of the colonic inflammatory stranding (Figure 4).

She was transferred to a tertiary medical center for surgical evaluation. She continued to have 10/10 diffuse abdominal pain and distention. Physical exam showed normal vitals with diffuse abdominal pain, no rebounding or guarding, and diffuse abdominal distention with no obvious fluid wave or shifting dullness. Laboratory tests showed total bilirubin 0.7 mg/dL, INR 1.2, and albumin 3.0 g/dL, liver enzymes ALP 143 U/L, AST 20 IU/L, ALT 6 IU/L, lipase 469 units/mg, hemoglobin 11.5 g/dL, WBC 19 × 10^9^/L, and PLT 265 × 10^9^/L.

Colorectal surgery was consulted and identified neither pericolonic abscesses nor an intraluminal obstruction. A repeat colonoscopy was performed at our institution for colonic decompression and inspection of the splenic flexure for possible intraluminal obstruction. Colonoscopy demonstrated increased luminal diameter in the descending and sigmoid colon without visible signs of mechanical obstruction or fistula near the splenic flexure. Several pedunculated polyps and serrated polyps were identified, but no evidence of colorectal cancer was present. After colonoscopy she again reported decrease in abdominal pain and distention but returned 48 hours after colonoscopy.

**Figure 5:**
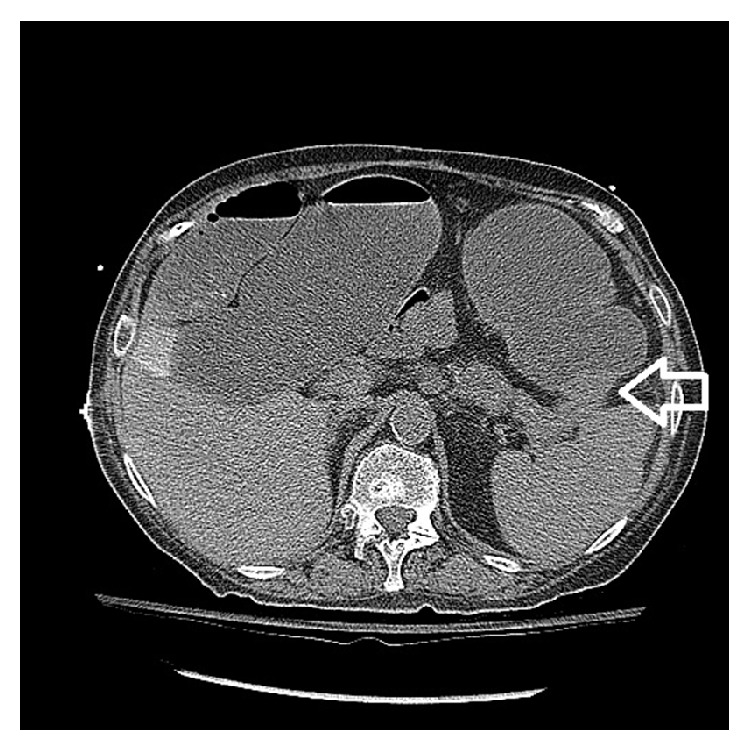
CT abdominal/pelvis obtained after repeat colonoscopy negative for intraluminal obstruction and return of abdominal distention and increase of abdominal pain. Question of pancreatic colonic fistula.

**Figure 6:**
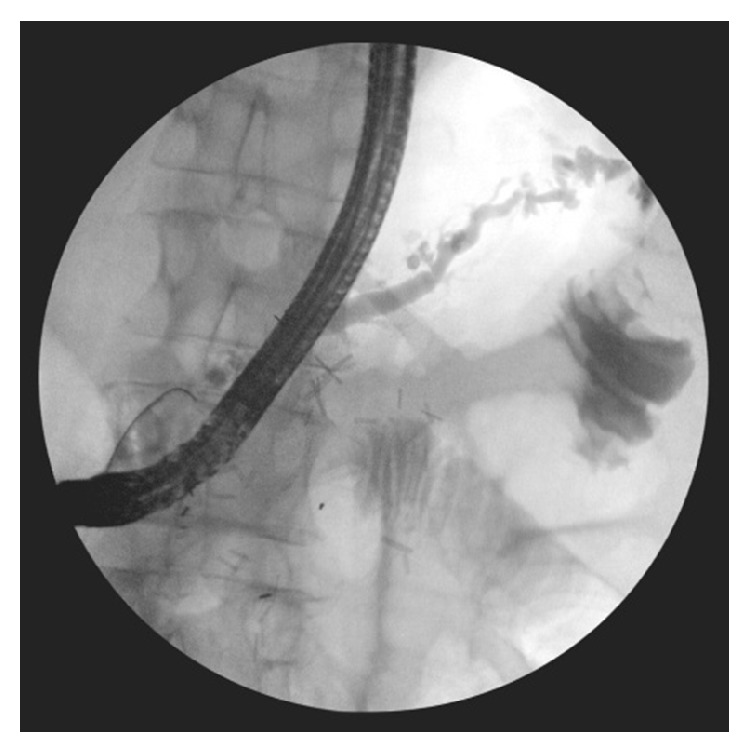
Pancreatogram demonstrating pancreatic colonic fistula.

**Figure 7:**
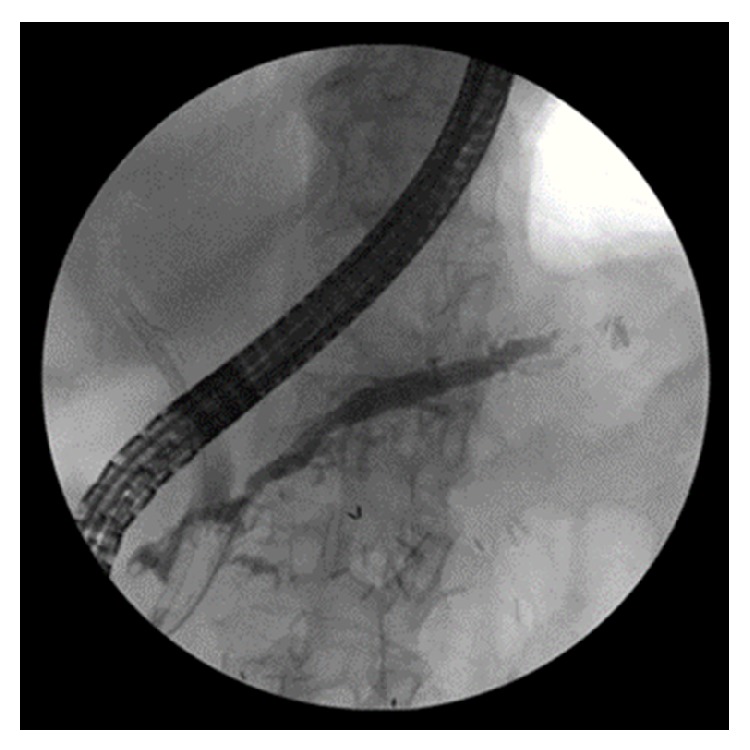
Pancreatogram after removal of 7F × 12 cm pigtail plastic stent in place for 3 months with resolution of the pancreatic colonic fistula.

Secondary to CT imaging showing fluid collections at the tail of the pancreas with extension near the splenic flexure endoscopic retrograde cholangiopancreatography (ERCP) was performed to further evaluate the pancreatic duct (Figure 5). The pancreatogram demonstrated tortuosity of the pancreatic duct with extravasation of contrast from the tail of the pancreas into the colon confirming a pancreatic colonic fistula (Figure 6). A single 7-French 12 cm plastic stent was placed in the pancreatic duct with subsequent improvement of abdominal pain and abdominal distention. She continued to improve and had return of her bowel function. On follow-up at 3 months she continued to have no abdominal pain or distention. Repeat ERCP at 3 months confirmed resolution of the pancreatic colonic fistula (Figure 7).

## 3. Discussion

PCF incidence ranges 0.4–5% with symptoms typically including diarrhea, hematochezia, fever, and abdominal pain [1, 2]. Hematochezia is the most common clinical manifestation found in 60% of patients and is associated with mortality rate of 77% [3]. Obstruction accounts for less than 1% [1].

Majority of PCF formation occurs in association with walled off pancreatic necrosis or abscess. WOPN or abscess causes destruction of the pancreatic parenchyma with release of digestive enzymes into the surrounding peritoneum causing fistula tract formation. Formation occurs days to month after acute pancreatitis. Discovery of PCF is usually after percutaneous catheter placement for drainage of WOPN or abscess with “tube checking” via fluoroscopy demonstrating contrast flow into the colon via the fistula tract. Surgical debridement of WOPN with subsequent identification has also been described. PCF not associated with WOPN or abscess is believed to be secondary to extension of necrosis into the transverse colon or pseudocyst that expands and applies pressure to the transverse colon causing erosion with tract formation.

In a study from Mayo [4] clinic 8 patients were identified with PCF formation of which 1 (12%) was spontaneous and 7 formed after debridement of pancreatic necrosis at 4 to 60 days. Identification can be difficult due to nonspecific symptoms (nausea, vomiting, and abdominal pain) and the insidious nature of PCF leading to diagnosis several months after initial episode of pancreatitis. Enhanced CT Imaging may show air fluid levels in a necrotic pancreas or directly visualize a fistula tract to the colon. However, CT may demonstrate obstruction or bowel wall thickening which may be interpreted as mechanical obstruction or colitis. High clinical suspicion for PCF warrants an investigation with ERCP to evaluate contrast extravasation into the colon along a fistula tract. Kochhar et al. [1] identified 4 patients with PCF via ERCP or colonoscopy 12 to 20 days after an episode of pancreatitis. Only 1 of these 4 patients presented with large bowel obstruction identified via CT imaging with dilated ascending colon.

Conservative treatment for PCF with bowel rest and antibiotics is associated with a 50% mortality if sepsis or hemorrhagic transformation occurs [5]. Surgical treatment with laparotomy or open repair improves mortality to 15%. Heeter et al. [3] identified 20 patients with PCF treated with antibiotics, percutaneous drainage, and pancreatic duct stent, but this was associated with abscess or WOPN as the source of PCF. Several other small case series have data showing resolution of spontaneous PCF with endoscopic clips being placed in the pancreatic duct or in the colon [6–8]. Limited case reports of treatment of PCF with pancreatic stents alone are effective [5].

Mrs. L case was unique in that her PCF was secondary to recurrent pancreatitis and extension of a pseudocyst in the tail of the pancreas into the transverse colon causing erosion and fistula tract formation. Lacking association with WOPN or abscess allowed conservative treatment with pancreatic stent placement. Resolution of the fistula at 3 months was demonstrated with no hemorrhagic or septic conversion.

PCF can present as large bowel obstruction. Patients with resolved acute pancreatitis who have radiographic evidence of splenic flexure obstruction, but without evidence of mechanical obstruction on colonoscopy, should be considered for ERCP to evaluate for PCF. PCF not associated with WOPN or peritoneal abscess can be treated conservatively with pancreatic duct stenting.
